# Effects of High-Temperature Treatments in Inert Atmosphere on 4H-SiC Substrates and Epitaxial Layers

**DOI:** 10.3390/ma17235761

**Published:** 2024-11-25

**Authors:** Francesca Migliore, Marco Cannas, Franco Mario Gelardi, Filippo Pasquali, Andrea Brischetto, Daniele Vecchio, Massimo Davide Pirnaci, Simonpietro Agnello

**Affiliations:** 1 Department of Physics and Chemistry Emilio Segrè, University of Palermo, Via Archirafi 36, 90123 Palermo, Italy; francesca.migliore02@unipa.it (F.M.); marco.cannas@unipa.it (M.C.); franco.gelardi@unipa.it (F.M.G.); 2 STMicroelectronics, Stradale Primosole, 95121 Catania, Italy; 3 ATeN Center, University of Palermo, Viale delle Scienze Ed. 18, 90128 Palermo, Italy

**Keywords:** 4H-SiC, thermal treatments, micro-Raman spectroscopy, graphitization, exciton recombination, time-resolved photoluminescence spectroscopy

## Abstract

Silicon carbide is a wide-bandgap semiconductor useful in a new class of power devices in the emerging area of high-temperature and high-voltage electronics. The diffusion of SiC devices is strictly related to the growth of high-quality substrates and epitaxial layers involving high-temperature treatment processing. In this work, we studied the thermal stability of substrates of 4H-SiC in an inert atmosphere in the range 1600–2000 °C. Micro-Raman spectroscopy characterization revealed that the thermal treatments induced inhomogeneity in the wafer surface related to a graphitization process starting from 1650 °C. It was also found that the graphitization influences the epitaxial layer successively grown on the wafer substrate, and in particular, by time-resolved photoluminescence spectroscopy it was found that graphitization-induced defectiveness is responsible for the reduction of the carrier recombination lifetime.

## 1. Introduction

The interest in developing devices for applications in emerging fields such as high-voltage and high-temperature electronics is pushing the use of wide-bandgap semiconductors. Among them, silicon carbide stands out for its excellent physical properties; in particular, the polytype 4H-SiC, which has a bandgap value of 3.3 eV, has a three-times-higher thermal conductivity and a ten-times-higher breakdown electric field strength than silicon. This brings two significant advantages for electronic applications. First, devices made of SiC with the same dimensions as Si devices withstand ten-times-higher voltages before breakdown. Additionally, it is possible to produce SiC devices that operate at the same voltage as Si devices but that are ten times smaller. Therefore, the strong potentiality of SiC for the production of high-power devices is evident. SiC also exhibits high chemical inertness, mechanical strength, high conductivity along a direction, and high-saturation drift velocity. Additionally, it is easy to control either n-type or p-type doping during the growth of both substrates and epitaxial layers. Uniquely, SiC is the only compound semiconductor with SiO_2_ as a native oxide, identical to that of silicon [1,2,3,4]. From an applicative perspective, it is important to highlight that SiC exhibits high radiation hardness due to its large threshold energy for defect formation. For this reason, SiC stands out as one of the most promising semiconductors to make devices that are able to operate in harsh environments and extreme conditions such as high levels of radiation, elevated temperatures, and environments with high chemical reactivity [5,6,7,8,9,10,11,12,13,14,15,16,17,18,19].

To fabricate a semiconductor device in 4H-SiC, it is generally necessary to grow an epitaxial layer with a specific thickness and doping level on a degenerate doped 4H-SiC substrate. This growth process, typically carried out via Chemical Vapor Deposition (CVD), involves high-temperature treatments that can reach up to 2000 °C [2,20,21]. Notably, it has been observed that thermal treatments in the 1200–1900 °C range, conducted in various atmospheres or ultra-high vacuum, can result in the formation of graphene layers on the surface of hexagonal or cubic SiC polytypes [22,23,24,25,26]. This highlights the potential vulnerability of the material to thermal treatments under different conditions, even during the epitaxial layer growth process. Consequently, the thermal stability of 4H-SiC at high temperatures remains a critical factor in the production of SiC devices.

In this study, we examined the stability of 4H-SiC substrates in an inert atmosphere within the temperature range of 1600–2000 °C, analyzing the wafer surface using micro-Raman spectroscopy. Traces of graphitization were observed on the thermally treated substrates. To evaluate the impact of this graphitization on subsequent epitaxial layer growth, a 12 μm thick epitaxial layer with n-type doping of 10^16^ cm^−3^ was grown on a treated substrate. The influence of graphitization in the epitaxial layer was investigated using time-resolved photoluminescence spectroscopy, focusing on the lifetime of the exciton recombination band.

## 2. Materials and Methods

STMicroelectronics (Catania, Italy) provided two substrates, named A and B hereafter, of 4H-SiC of 6-inch and 350 μm thickness. The substrates have the same nominal features of doping (n-type 10^19^ cm^−3^ realized using nitrogen) and thickness. They differ, however, in the extent of the facet region, a darker area on the wafer associated with substrate growth, which has higher doping than the rest of the wafer. Specifically, sample A has a circular facet, 9 cm in diameter, at the center of the wafer, while in sample B, the facet extends across the entire wafer. Both substrates, A and B, underwent thermal treatment within the temperature range of 1600–2000 °C. This process involved a rapid thermal ramp-up to the target temperature (below 2000 °C), a plateau of a few seconds at the peak temperature in Ar atmosphere, followed by natural cooling (approximately 3 h). Additionally, STMicroelectronics grew a 12 μm thick n-type 4H-SiC epitaxial layer with doping of 10^16^ cm^−3^ on two new thermally treated substrates.

The entire surfaces of the samples were characterized before and after the thermal process using micro-Raman spectroscopy to obtain information on crystal structure and electronic properties. Spectra were collected in back-scattering geometry with a Horiba HR-Evolution Micro-Raman system, equipped with a confocal microscope (100× magnification) and a laser excitation wavelength of 633 nm, filtered with a neutral density (ND) filter at 50% to prevent sample damage. The pinhole size was set to 200 or 50 μm for measurements on substrates and epitaxial layers, respectively. All spectra were acquired using a grating with 600 lines/mm and calibrated to the Si peak at 520.7 cm^−1^. Notably, the system’s vertical resolution is 2–3 μm, ensuring that measurements on epitaxial layers were unaffected by the underlying substrate.

The exciton recombination band lifetime at 3.2 eV (associated with nitrogen doping) was investigated through time-resolved photoluminescence spectroscopy, using excitation at 4.66 eV (266 nm). The penetration depth of the selected excitation wavelength (around 0.6 μm) enabled the generation of exciton pairs primarily within the epitaxial layer. Measurements were conducted with a tunable laser system consisting of an optical parametric oscillator (OPO) pumped by the third harmonic of a pulsed, Q-switched Nd:YAG laser, producing 5 ns pulses at a 10 Hz repetition rate. Emission spectra were collected with an intensified CCD camera at progressively increasing delays from the laser pulse and within a defined time window.

## 3. Results and Discussion

**Figure 1:**
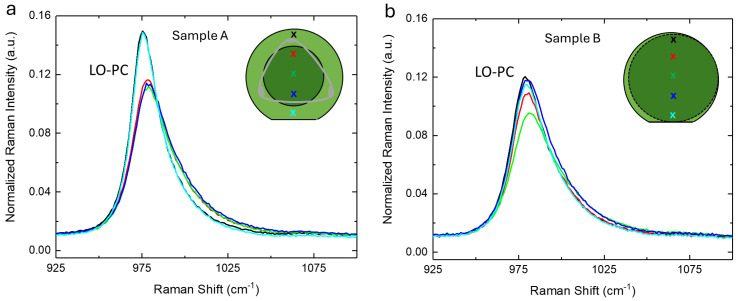
(**a**) Normalized micro−Raman spectra of substrate A along the diameter in the range 925–1100 cm^−1^. (**b**) Normalized micro−Raman spectra of substrate B along the diameter in the range 925–1100 cm^−1^. The spectra are normalized to the TO band at 780 cm^−1^ amplitude. The graphical representations of the substrates are inserted in both panels. The color of the spectra refer to the positions where they were acquired, which are marked by an x of the same color on the graphical representation.

The micro-Raman spectra of substrates A and B in the range of the LO-PC (longitudinal optical phonon–plasmon coupled) band (925–1100 cm^−1^) are reported, respectively, in Figure 1a,b together with a graphical representation of the samples themselves. The LOPC asymmetric shape is well-known in the literature and is related to doping concentration. In fact, as in other polar semiconductors, the free carriers interact with the LO phonons, giving rise to the longitudinal optical phonon–plasmon coupled mode. As a consequence, this Raman mode is strongly affected by the carrier density. In particular, when the carrier density increases, the LO peak shifts toward high Raman shift values and features peak broadening and asymmetry (LOPC) [21,27,28]. In sample A, it is evident that the doping in the facet and in the rest of the wafer is different, in particular, in the facet, the doping is higher. Instead, in sample B, the doping is quite homogeneous (reasonably, because the facet is extended across the whole wafer). It is worth noting that based on the spectral characteristics of the Raman LOPC band, the doping of sample B is the same as sample A in the facet area.

**Figure 2:**
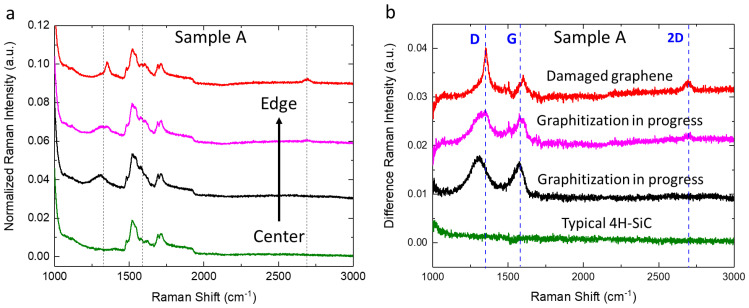
(**a**) Normalized micro−Raman spectra, with respect to TO amplitude band, of substrate A in different radial positions subsequent to thermal treatment at 1650 °C in the range of the second-order Raman peaks. Specifically, the green spectrum corresponds to a circular area with a 3 cm diameter centered on the wafer, the pink spectrum to positions 1–2 cm from the edges, the red spectrum to the edges, and the black spectrum to the remaining surface area. Dashed lines are reported as guides for the eye to mark relevant spectral positions. (**b**) Normalized micro-Raman spectra of Figure 2a after the subtraction of the reference spectrum of 4H-SiC. The dashed lines labeled D, G, and 2D indicate the Raman peaks associated with graphene.

In Figure 2a, micro-Raman spectra acquired in different radial positions, from the center to the edges, of sample A after the thermal process conducted at 1650 °C are shown. Each color refers to a particular area of the wafer: green for a circular area 3 cm in diameter centered at the center of the wafer; pink at 1–2 cm from the edges; red on the edges; and black on the remaining parts of the surface. It is evident that the surface of sample A is not homogeneous after the thermal process. In particular, the spectra are characterized by the typical bands of 4H-SiC together with specific shape changes (along the black dashed lines, as a guide for the eye) going from the center to the edges of the wafer [29,30].

To demonstrate the effects of the thermal treatment, the spectra of Figure 2a after the subtraction of the reference Raman spectrum of the same kind of substrate of 4H-SiC before any treatment are reported in Figure 2b. Furthermore, with blue dashed lines, the typical Raman bands D (∼1350 cm^−1^), G (∼1580 cm^−1^), and 2D (∼2700 cm^−1^) of graphene are indicated [31,32]. The green spectrum, collected in the center of sample A, has no difference in signal that would indicate that the corresponding part of the wafer is made of high quality, unmodified 4H-SiC. In the black spectrum, which refers to the most widespread region of the wafer, two bands around 1300 cm^−1^ and 1580 cm^−1^ are visible. In the pink spectrum, at 1–2 cm from the edges, it is possible to observe a shift in the bands toward high Raman shift values. Moreover, even if it is hardly visible for the magnification motif, a band around 2700 cm^−1^ is present. Lastly, in correspondence with the edges, for the red spectrum, the band around 2700 cm^−1^ is evident and the bands at 1350 cm^−1^ and 1600 cm^−1^ become narrower. The red spectrum matches with the Raman spectra of low-quality and damaged graphene, whereas the other spectra resemble those of amorphous carbon [29,31,33].

**Figure 3:**
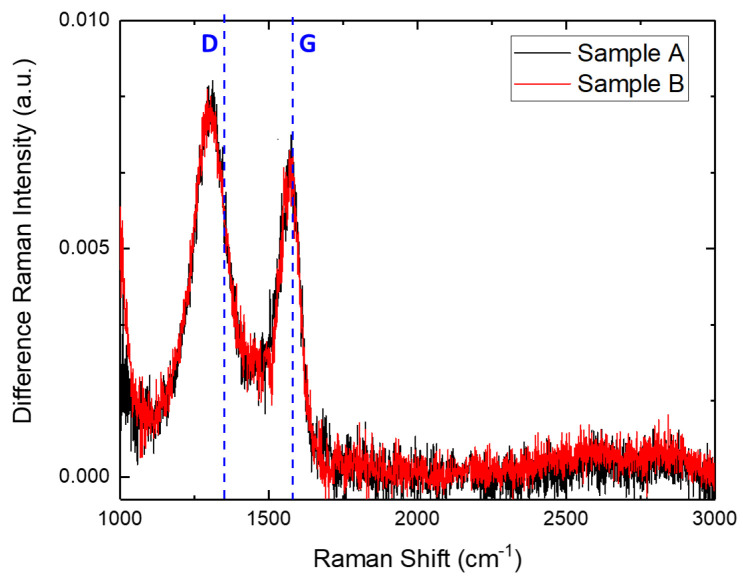
Normalized micro-Raman spectra, with respect to TO amplitude band, in the range of the second-order Raman peaks of sample A (black) and sample B (red) after the subtraction of the 4H-SiC reference spectrum. The spectra were acquired from positions with the same doping level. The dashed lines labeled D and G indicate the Raman peaks associated with graphene.

The micro-Raman characterization conducted on sample B after the thermal process (data not reported) indicates that the effect of the treatment is homogeneous all over the wafer. To demonstrated these effects, in Figure 3, the recorded micro-Raman spectrum after the subtraction of the reference spectrum is reported in red. Furthermore, in this case, two bands at 1300 cm^−1^ and 1580 cm^−1^ related to amorphous carbon are evident. In addition, in Figure 3, the most common micro-Raman difference in spectrum of sample A is reported in black. It is evident that this one is similar to the spectra of sample B after the thermal treatment and the subtraction of the reference 4H-SiC spectrum. It is worth noting that the areas of sample A and sample B compared in Figure 3 have the same doping. For this reason, the observed effects seem to be related to the doping levels of the material.

It is possible to explain the observed results as a graphitization process of the surface triggered by the desorption of Si from the SiC lattice and the subsequent reorganization of the C atoms. This is supported by the fact that this mechanism is exploited to produce high-quality graphene on 4H-SiC or on other SiC polytypes with hexagonal symmetry in an inert atmosphere or in vacuum starting from around 1000 °C up to 2000 °C (depending on other parameters such as gas pressure and growth time, which can reach 30 min) [22,23,24,25,26,31,34]. In this study, the substrates were treated for just a few seconds at 1650 °C, and this was sufficient to start the graphitization of the wafers. Therefore, this short treatment time range can explain the almost total presence of amorphous carbon on both sample A and sample B.

**Figure 4:**
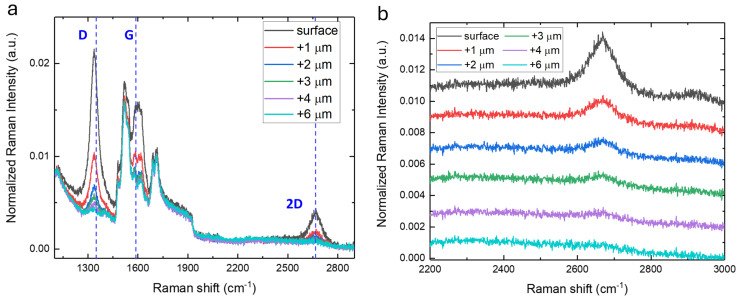
(**a**) Micro−Raman measurements in the range of the second−order Raman peaks, normalized with respect to the TO amplitude band, acquired on a point of the edge of sample A, varying the z-positions underneath the sample surface. (**b**) Zoom in the 2200–3000 cm^−1^ range of the spectra in panel (**a**). The spectra are vertically shifted for convenience.

To further investigate the graphitized layer, a scan of the region underneath the surface of the substrate (z-profile) was carried out by micro-Raman spectroscopy. In particular, in Figure 4, the z-profile in a point of the edge of sample A (red spectrum in Figure 2a) is reported. Moving beneath the surface of the sample, the spectrum changes gradually until a depth of 4 μm below the surface, where the traces of graphitization completely disappear. Since the estimated vertical resolution of the micro-Raman setup is 2–3 μm, it is possible to conclude that the effect induced by the thermal treatment is limited to the surface of the sample within 3 μm of the surface.

**Figure 5:**
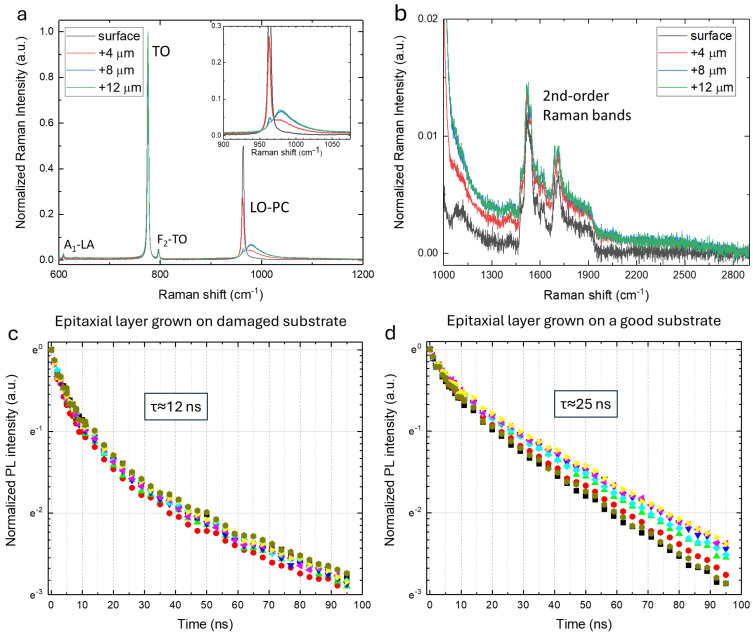
(**a**) Micro-Raman measurements, normalized with respect to the TO amplitude band, acquired at different positions underneath the epitaxial layer surface grown on one of the thermally treated damaged substrates. The spectrum collected at 12 μm is in correspondence with the interface between the substrate and epitaxial layer, and the inset reports a zoom of the LOPC band. (**b**) Zoom of the 1000–2900 cm^−1^ range of the spectra in panel (**a**). (**c**) Photoluminescence time decay (excitation wavelength 266 nm) of the excitonic band recorded at different positions (reported with different colors) on the epitaxial layer grown on the damaged substrates. (**d**) Photoluminescence time decay (excitation wavelength 266 nm) of the excitonic band recorded at different positions (reported with different colors) on the epitaxial layer grown on a high-quality substrate.

To study how the graphitization revealed on the thermally treated substrates can affect the subsequent step of the device growth, an epitaxial layer of 12 μm thickness and n-type doping of 10^16^ cm^−3^ was grown on two new substrates, which presented the graphitization effects described above. In particular, the structure and the doping (qualitatively) of the wafers after the growth of the epitaxial layer were monitored by micro-Raman spectroscopy from the surface of the epitaxial layer down to the interface with the substrate, performing the z-profile reported in Figure 5a,b. It is possible to notice that no traces of graphitization are visible even at a depth of 12 μm below the surface, that is, at the interface between the substrate and the epitaxial layer. This indicates that the graphitized layer on the substrate does not affect the crystal structure of the epitaxial layers grown on it.

To further explore the effects of the graphitization of the substrate once an epitaxial layer is grown on it, time-resolved photoluminescence spectroscopy measurements were conducted on the epitaxial layer grown on a damaged substrate to study the lifetime of the excitonic band (at around 3.2 eV). In fact, the carrier lifetime is one useful parameter to evaluate the quality of semiconductor materials, and time-resolved photoluminescence spectroscopy can be used as a probe of the epitaxial layer quality due to its sensitivity to the presence of point defects and the possibility to obtain (qualitatively, by comparison) local information about their presence [35]. The normalized photoluminescence time decay of the excitonic band recorded in different positions on the epitaxial layer grown on the two damaged substrates and on an epitaxial layer grown (under the same conditions) on a high-quality substrate are, respectively, reported in Figure 5c,d. In this study, the lifetime is defined as the time required for the intensity of the excitonic band to decay to 1/e of its value measured 6 ns after the laser pulse peak, and the associated errors are related to the experimental uncertainty arising from the repeatability of the measurements. The lifetime values are different, in particular, they correspond to 12 ± 2 ns in the epitaxial layer grown on the damaged substrates and 25 ± 2 ns in the epitaxial layer grown on the high-quality substrate. Considering that the utilized substrates have the same nominal features (thickness, doping, amount of extended defects); that the epitaxial layers were grown in exactly the same way; that the crystal quality of the epitaxial layer seems to be not affected by the graphitization of the substrate underneath; and that a shorter lifetime is associated with an increase in the number of defects that influence the exciton recombination, our results demonstrate that a remembrance of the graphitization process is present even if the carbon-related species have been removed or masked during the growth of the epitaxial layer.

## 4. Conclusions

The thermal stability in an Ar atmosphere at a temperature in the range 1600–2000 °C of 4H-SiC substrates was studied. Amorphous carbon and traces of damaged graphene were observed by micro-Raman spectroscopy after the thermal treatment. Furthermore a dependence on doping was observed. The effect of graphitization on the surface of the substrates is attributed to the desorption of Si from the 4H-SiC lattice. The epitaxial layer grown on thermally treated damaged substrates was studied by micro-Raman and time-resolved photoluminescence spectroscopy. The crystal structure of the epitaxial layer was not affected by the graphitization damages of the substrate underneath, but despite that, it has an important influence on the recombination lifetime of the excitonic couple, shortening it.

## Data Availability

The original contributions presented in this study are included in the article. Further inquiries can be directed to the corresponding author.
